# A systematic review on antibiotic therapy of cutaneous bacillary angiomatosis not related to major immunocompromising conditions: from pathogenesis to treatment

**DOI:** 10.1186/s12879-024-09253-9

**Published:** 2024-04-08

**Authors:** Salvatore Rotundo, Maria Teresa Tassone, Nadia Marascio, Helen Linda Morrone, Simona Gigliotti, Angela Quirino, Alessandro Russo, Giovanni Matera, Enrico Maria Trecarichi, Carlo Torti

**Affiliations:** 1 Dipartimento di Scienze Mediche e Chirurgiche, Università “Magna Graecia”, Catanzaro, Italy; 2 Dipartimento di Scienze della Vita, Unità Operativa Complessa di Microbiologica Clinica, Università “Magna Graecia”, Catanzaro, Italy; 3 Unità Operativa Complessa di Malattie Infettive e Tropicali, Azienda Ospedaliero-Universitaria “R. Dulbecco”, Catanzaro, Italy; 4 Dipartimento di Scienze di Laboratorio e Infettivologiche, Fondazione Policlinico Universitario Agostino Gemelli IRCCS, Rome, Italy; 5 Dipartimento di Sicurezza e Bioetica, Università Cattolica del Sacro Cuore, Rome, Italy

**Keywords:** *Bartonella*, Antibiotic, Bacillary angiomatosis, Emerging disease, PCR, One health

## Abstract

**Background:**

Cutaneous bacillary angiomatosis (cBA) is a vascular proliferative disorder due to *Bartonella* spp. that mostly affects people living with HIV (PLWH), transplanted patients and those taking immunosuppressive drugs. Since cBA is mostly related to these major immunocompromising conditions (i.e., T-cell count impairment), it is considered rare in relatively immunocompetent patients and could be underdiagnosed in them. Moreover, antimicrobial treatment in this population has not been previously investigated.

**Methods:**

We searched the databases PubMed, Google Scholar, Scopus, OpenAIRE and ScienceDirect by screening articles whose title included the keywords “bacillary” AND “angiomatosis” and included case reports about patients not suffering from major immunocompromising conditions to provide insights about antibiotic treatments and their duration.

**Results:**

Twenty-two cases of cBA not related to major immunocompromising conditions were retrieved. Antibiotic treatment duration was shorter in patients with single cBA lesion than in patients with multiple lesions, including in most cases macrolides and tetracyclines.

**Conclusions:**

cBA is an emerging manifestation of *Bartonella* spp. infection in people not suffering from major immunocompromising conditions. Until evidence-based guidelines are available, molecular tests together with severity and extension of the disease can be useful to personalize the type of treatment and its duration.

## Background

*Bartonella* spp. includes fastidious and arthropod-borne Gram-negative bacilli infecting both vector insects and mammalian hosts [1]. Several animals act as a reservoir since they are often asymptomatic, while fleas and other blood-sucking arthropods are the vectors of bartonellosis [2, 3]. Although many *Bartonella* spp. have been detected in both wild animals and pets, few data have been published regarding transmission of these bacteria in pets and wild animals and the possible pathogenicity for these hosts [4–7]. The most studied species in human pathology are *B. henselae, B. bacilliformis* and *B. quintana* [8]. *B. bacilliformis* is responsible of Carrion’s disease, which stands among the array of neglected tropical diseases, often overlooked despite its significant impact on affected communities. The initial acute phase of Carrion’s disease manifests with fever and haemolytic anaemia, presenting a formidable mortality rate ranging from 44 to 88% in untreated individuals [9]. Following this acute stage, a subsequent phase ensues, which may emerge weeks to months later, often with or without a history of antecedent illness. This phase is characterized by the eruption of clusters of skin lesions, categorized as nodular lesions, called verruga peruviana [9, 10] since this disease primarily affects regions within the Andean cordillera spanning across Peru, Ecuador, and Colombia. This geographical confinement is largely attributed to the behavior of its suspected primary vector, *Lutzomyia verrucarum*, characterized by its limited, hopping flight capabilities and sensitivity to extreme temperatures [9]. The pathological manifestations of *Bartonella* spp. infections are widely heterogeneous, including asymptomatic bacteremia, neurological disorders, myocarditis, retinitis, chronic lymphadenopathies, endocarditis, sepsis, and vascular proliferative disorders such as bacillary peliosis and cutaneous bacillary angiomatosis (cBA) [11, 12], which are caused by *B. henselae* or *B. quintana* [11, 13–22]. However, cBA and verruga peruana are difficult to distinguish since those are nearly identical presenting as angiomatous lesions [23]. Homelessness, low socioeconomic status and being infested with lice are the most common risk factors associated with cBA caused by *B. quintana*, while owning a cat bearing fleas or cat bites and scratches are associated with cBA due to *B. henselae* [11, 13]. Regarding the host, cBA is mostly described in severely immunocompromised patients with T-cell response impairment such as transplant recipients, those taking immunosuppressive drugs and people living with HIV (PLWH). Only for the latter cathegory treatment guidelines are available, issued by the National Institute of Health (NIH) that recommends using doxycycline or erythromycin for at least three months [24]. However, cBA in patients not suffering from major immunocompromising conditions is considered rare [14] and there is no consensus about treatment, since no studies enrolled enough of these patients to validate type and duration of antimicrobic treatment. Therefore, we focused on patients not suffering from immunocompromising conditions and reviewed current literature to provide insights about antibiotic treatment and its duration.

## Methods

Two independent reviewers searched the database PubMed, Google Scholar, Scopus, OpenAIRE and ScienceDirect by screening articles whose title included the keywords “bacillary” and “angiomatosis”. Only case reports regarding cBA published until November 15th, 2023, written in English language and published in peer-reviewed journals dealing with patients without major immunocompromising conditions, either ongoing or in past medical history (i.e., PLWH, organ transplant recipient, affected by hematological malignancy and/or taking immunosuppressive drugs) were included. Since we were interested in correlating the patient clinical healing with the treatment provided, only cases with available type and duration of antibiotic therapy were included. References of each article were checked to include as many cases as possible regarding cBA not related to major immunocompromising conditions. The outcome was the clinical cure (i.e., healing of skin lesions).

**Fig. 1:**
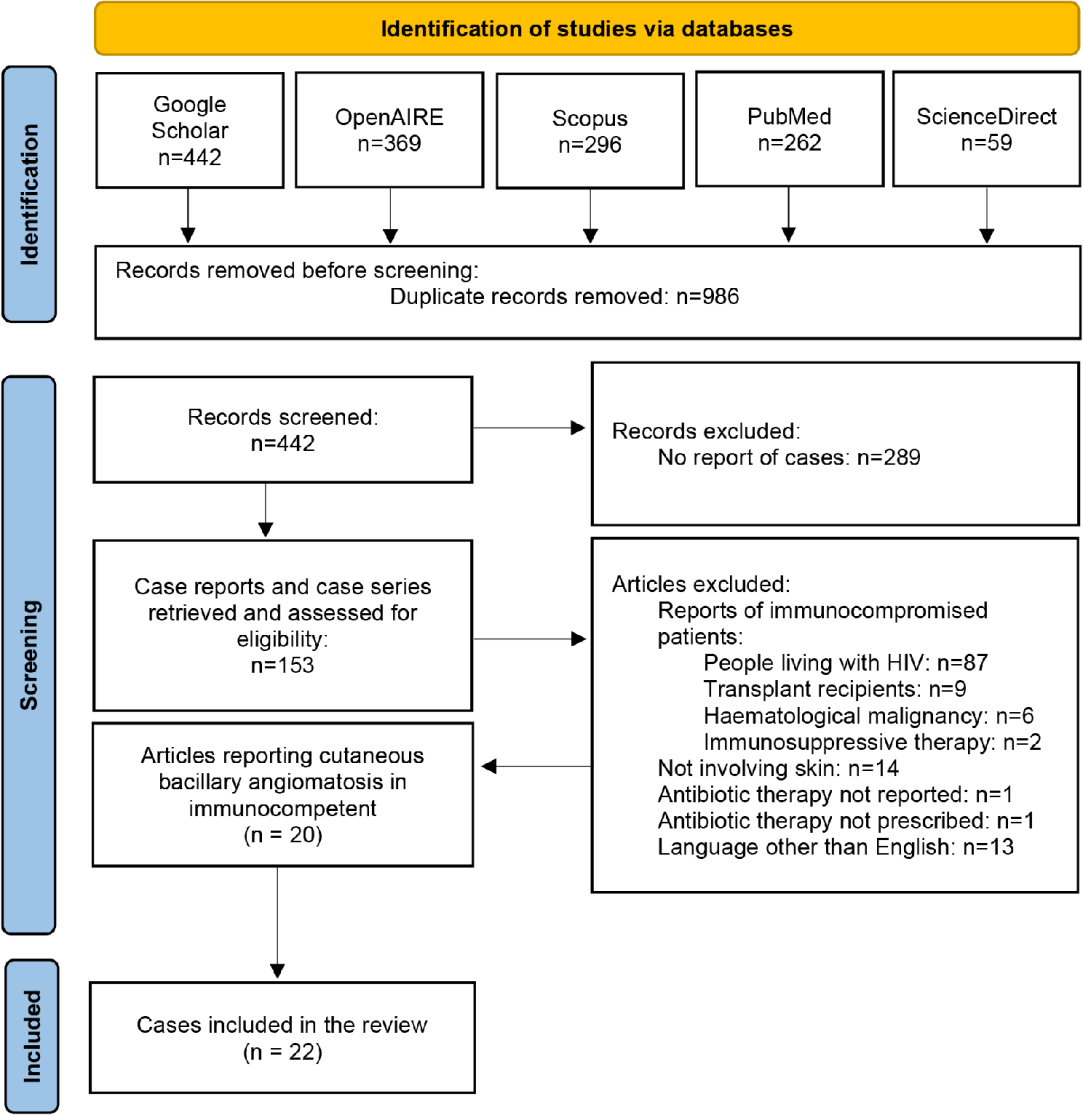
PRISMA flowchart of study selection process

The systematic review protocol was registered on PROSPERO on June 30th, 2023 (registration number: CRD42023437976). The protocol and article follow the PRISMA checklist for the reporting of systematic reviews [25] (Fig. 1).

## Results

Up to November 15th, 2023, 24 cases of cBA are reported in not severely immunocompromised hosts [8, 11, 14–22, 26–36]. In one case antibiotic treatment was not prescribed [31] and in another case therapy was not reported [35]. Therefore, 22 cases were included in this analysis whose characteristics are summarized in Table 1.

**Table 1:** Characteristics of the 22 cases included in this analysis. M: male; NR: not reported; F: female; PCR: polymerase chain reaction

Author, year of publication	Country	Patient age and sex	Risk factor(s)	Site of lesion(s)	Diagnostic method	Lesion(s)	Antibiotic (duration)
Cockerell CJ et al., 1990 [27]	United States of America	37 M	Parakeet	Forearm	Culture	Multiple	Erythromycin (3 months and 2 weeks)
Tappero JW et al., 1993 [22]	United States of America	41 NR	Cat, fleas, chiggers	Nose	Histopatholology	Single	Minocycline (4 to 6 weeks)
		74 NR	Cat, fleas, mites	Neck	PCR	Single	Erythromycin/ doxycycline* (4 to 6 weeks)
		42 NR	Cat, fleas, fire ants	Genital	PCR	Single	Erythromycin (4 to 6 weeks)
Paul MA et al., 1994 [29]	United States of America	6 F	Cat	Neck	Histopatholology	Single	Erythromycin (6 weeks)
Karakaş M et al., 2000 [16]	Turkey	21 F	Burn	Face	Histopatholology	Multiple	Erythromycin (2 months)
Gangopadhya AK et al., 2001 [26]	India	65 M	None	Forearm	Histopatholology	Multiple	Erythromycin (2 weeks)
Asharaf M et al., 2002 [18]	India	5 M	Trauma	Lips, knees, buttocks, ankles, elbows	Histopatholology	Multiple	Erythromycin (3 months)
Kayaselçuk F et al., 2002 [30]	Turkey	67 F	None	Scalp	Histopatholology	Single	Ciprofloxacin (10 days)
Karakas M et al., 2003 [17]	Turkey	32 M	None	Leg	Histopatholology	Multiple	Erythromycin (2 months)
Turgut M et al., 2004 [19]	Turkey	6 M	Trauma	Forehead	Histopatholology	Single	Clarithromycin plus rifampin (7 weeks)
Bernabeu-Wittel J et al., 2010 [8]	Spain	59 F	None	Ankle	PCR	Single	Doxycycline (2 months)
Kacar N et al., 2010 [32]	Turkey	10 M	Trauma	Leg	Histopatholology	Single	Erythromycin (1 week)
Bellissimo-Rodrigues F et al., 2010 [34]	Brazil	32 F	None	Thumb	Histopatholology	Single	Erythromycin (4 week)
Zarraga M et al., 2011 [11]	United States of America	10 F	Cat	Chest	Histopatholology	Single	Azithromycin (14 days)
Albayrak A et al., 2011 [20]	Turkey	5 M	None	Arm	Histopatholology	Single	Erythromycin (2 months and 2 weeks)
Blattner C et al., 2014 [33]	United States of America	76 F	None	Upper lip	Histopatholology	Single	Erythromycin/ doxycycline* (2 weeks)
Iraji F et al., 2015 [28]	Iran	26 F	None	Arm, fingers	Histopatholology	Multiple	Clarithromycin (3 months)
Nikam BP et al., 2018 [15]	India	45 F	Cat	Arm, forearm, ankle	Histopatholology	Multiple	Doxycycline (4 months)
Balaban M et al., 2019 [21]	Romania	43 M	Trauma	Face	Histopatholology	Multiple	Clarithromycin (6 weeks)
Agrawal S et al., 2022 [14]	India	45 M	None	Hands, forearm	Histopatholology	Multiple	Doxycycline (4 months)
Rotundo S et al., 2023 [36]	Italy	67 M	Cat	Forearm	PCR	Single	Doxycycline/ clarithromycin^§^ plus levofloxacin (1 month)

^*^Erythromycin was switched to doxycycline because the patient became intolerant

^§^Doxycycline was switched to clarithromycin due to side effect

Among these patients, seven were reported in the United States of America, six in Turkey, and four in India. Only one case for each country was reported in Spain, Brazil, Iran, Romania and Italy. Nine were females and ten were males, in three cases gender was not reported [22]. Six patients were children. The most common risk factor for cBA was having had a contact with a cat (7/22). Other possible risk factors were trauma (4/22), arthropod bite (3/22), burn (1/22) and having contact with a parakeet (1/22). Three patients reported multiple risk factors [22] while in nine cases no risk factors were identified. Nine patients had at least a lesion involving their head or neck and nine patients had their upper limbs involved. Nine cases showed multiple cBA lesions, while in thirteen cases patients had a single cBA lesion. In those instances reporting single cBA lesions, notable heterogeneity in size was observed, ranging from as small as 5 mm to as large as 10 cm. Diagnosis was mainly made by histopathology (17/22), polymerase chain reaction (PCR) was positive in four cases on biopsy and in one case [36] was reported to be positive even on blood sample. However, in many of the other cases it was not stated whether PCR was performed or not. Culture was reported to be positive in only one case [27].

In multiple cBA lesion, lesion removal was performed for diagnostic purposes. In single cBA lesions, complete removal was reported in five cases before antibiotic treatment [11, 19, 20, 29, 36]. Only in one case [30] it was specified that the lesion was biopsied without complete removal. In the remaining cases, biopsies were performed without specifying whether the lesion was completely removed or not [8, 32–34].

**Fig. 2:**
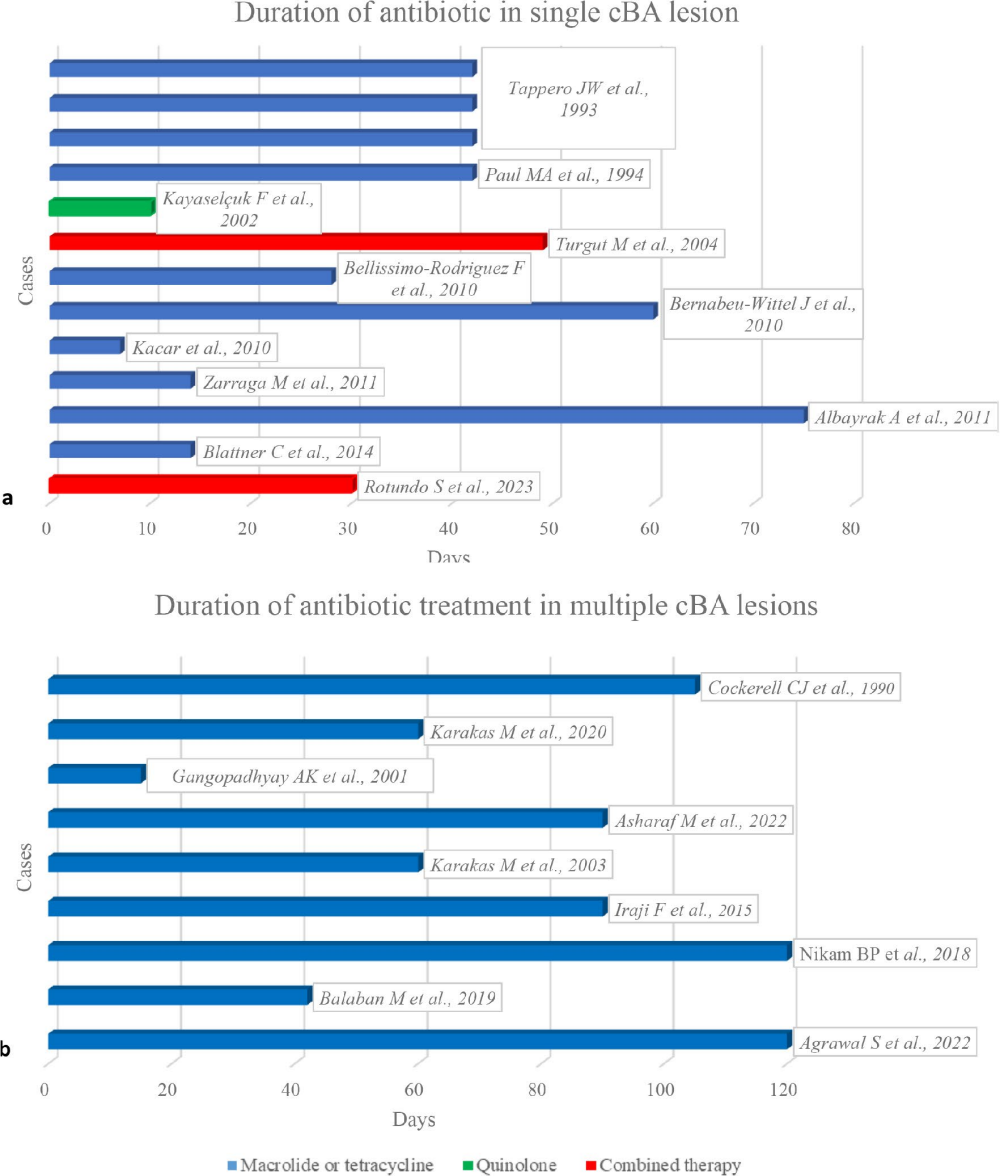
Type and duration of antibiotic treatment in single (**a**) and in multiple (**b**) cutaneous bacillary angiomatosis (cBA) lesions

Macrolides were used in most cases (17/22), being erythromycin the most frequently prescribed antibiotic (12/22) and only in two patients it was switched due to intolerance [22, 33]. Clarithromycin alone was used in three cases and in one case in association with rifampin [19]. One patient received azithromycin [11]. Tetracyclines were prescribed in seven cases: doxycycline was prescribed in six patients, including the two mentioned above who did not tolerate macrolides [22, 33]; in one patient doxycycline was used in a combined regimen and later switched due to side effect onset [36]. One patient was treated with minocycline [22]. Only one patient received a quinolone (ciprofloxacin) as monotherapy [30] (Fig. 2).

**Fig. 3:**
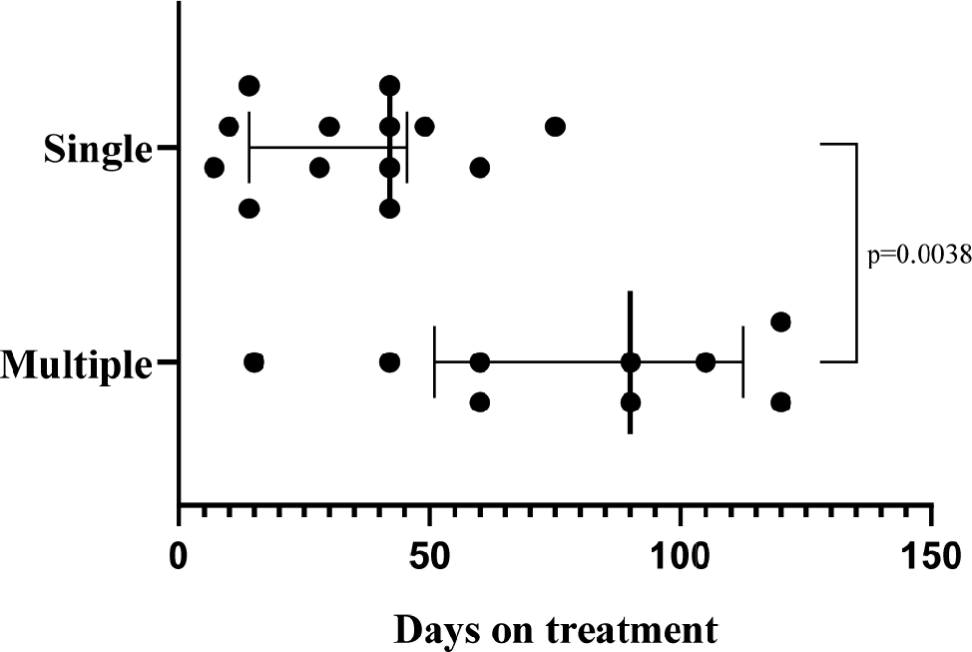
Comparison of duration of antibiotic therapy between single and multiple cutaneous bacillary angiomatosis. Statistical analysis was performed by GraphPad Prism 9.0 Version 9.3.1 (GraphPad Software, San Diego, CA 92,108), and the data are expressed as median ± interquartile range. Mann-Whitney test was applied to analyze duration of antibiotic therapy in the two groups. Exact duration of antibiotic therapy in patients reported by Tappero JW et al. [22] was not specified and ranged from four to six weeks. The maximum duration of antibiotic therapy (six weeks) was considered for this analysis

Antibiotic treatment duration was significantly (*p* = 0.0038, Fig. 3) shorter in patients with a single cBA lesion (median 42 days, interquartile range [IQR]: 14–45 days) than in those with multiple cBA lesions (median 90 days, IQR: 51–112 days).

## Discussion

In most cases included in the review (17/22), cBA affected body surfaces more susceptible to vector insect exposure which are not protected by clothing (such as head, neck and upper limbs). Indeed, despite *Bartonella* spp. is usually inoculated into the derma by a vector insect that feeds on blood [37], it was previously identified in non-blood-sucking arthropods such as *Dermatophagoides* spp [38]. and *Demodex* spp [39].. Interestingly, in 5/22 cases included in this review, burn or trauma were the only risk factors reported that could be associated with cBA. Therefore, we may hypothesize that these mites could cause both the transmission and the persistence of *Bartonella* spp. in cases in which skin is already damaged, since a bite from a blood-sucking arthropod may not necessarily be the only way of transmission of *Bartonella* spp. infection [38, 39] although no currently available data suggest that these arthropods are associated with *Bartonella* spp. infection in humans.

Once *B. henselae* or *B. quintana* reaches the skin, it eludes phagocytosis with mechanisms that are still being clarified [36, 40, 41]. For instance, *B. quintana* developed a low pathogenic lipopolysaccharide (LPS) which suppresses the classical activation of toll-like receptor (TLR)-4 preventing the production of acute phase proteins by peripheral blood mononuclear cells [42]. Moreover, *B. henselae* and *B. quintana* hide in mesenchymal staminal and endothelial cells and upregulate several angiogenic factors with both direct mitogenic and antiapoptotic properties (including chemokines, interleukins and the vascular endothelial growth factor) [21, 43, 44] which have a key role in developing angioproliferative lesions [37] and in escaping the immune system. Growth of these angioproliferative lesions depends on the persistence of bacteria in blood vessels and could be reverted by *Bartonella* spp. eradication [45]; indeed, some antibiotics demonstrated not only activity against the bacteria but also direct modulation of endothelial cell proliferation [46] Notably, *B. bacilliformis*, *B. henselae*, and *B. quintana* share similarities in producing angiogenic factors, suggesting a common mechanism underlying endothelial proliferation [10]. The main host factor involved in hampering cBA appears to be the T-cell response [47]. Indeed, T-cells play a pivotal role in controlling *Bartonella* spp. infection since these cells support a Th-1 response and activate macrophages in infections due to *Bartonella* spp [10].. Moreover, since the first description reported 40 years ago in a black man with T CD4 + cell count below 200/µL [48], immunocompromised patients with T-cell response impairment (i.e., PLWH [49–58], organ transplant recipients [59–69], those on immunosuppressive medication [70–72]) were found to be most at risk of developing cBA due to *B. henselae* or *B. quintana*. Despite in some cases retrieved for this analysis T CD4 + cell count was reported to be normal [8, 22, 28, 31, 36], we can speculate that in these patients a functional T-cell impairment could explain some of these rare occurrences. For instance, the patient reported by Kaçar N. et al. was affected by chronic HBV infection [32] and it has been shown that HBV leads to a T-cell functional impairment characterized by compromised cytokine production and upregulation of multiple inhibitory receptors [73]. Moreover, immunosenescence (i.e., age-related changes that affect T-cell capacity to respond to infections [74]) could impair T-cell functions in elderly patients included in this review [22, 26, 30, 33, 36]. Therefore, although bartonellosis is classically considered as a disease affecting immunocompromised hosts, patients not suffering from major immunocompromising conditions may also be affected by cBA, as emerges from this review.

The complex and not completely understood interactions between host T-cell responses and *Bartonella* spp. virulence factors mentioned above lead to the red to violaceous lesions which are clinically seen even in not severely immunocompromised patients with cBA and the typical granulomatous and angioproliferative lesions of cBA which can be observed on microscopic examination [8, 11, 14–22, 26–33, 36]. In such cases, diagnosis could be very tricky for several reasons. First, physicians do not include cBA in differential diagnosis since it is considered an infectious disease affecting only severely immunocompromising patients. Second, cBA lesions can be mistaken for similar ones due to both infective (e.g., *Mycobacterium* spp., *Nocardia* spp., *Sporothrix* spp., *Histoplasma* spp.) or other (e.g., neoplasm, trauma) causes [8, 11, 14, 15]. Third, *Bartonella* spp. are seldom isolated from cutaneous specimens since they are difficult to culture [8]. Fourth, PCR is not widely available in all settings. Indeed, among the cases we identified, it was performed only in three patients to diagnose cBA [8, 22]. For all these reasons, cBA could be underdiagnosed [3, 14] thus it is likely that its prevalence is underestimated, although an increase in incidence should be expected in the next years [75].

Antibiotic therapy is the mainstay treatment for cBA and different schemes were used. Several drugs such as macrolides, aminoglycosides, rifampin, ciprofloxacin and β-lactam antibiotics are active against *Bartonella* spp [12, 21].. Erythromycin is considered the first choice in PLWH and, even if more studies are needed to support this indication, in this analysis its effectiveness is likewise confirmed in patients without major immunocompromising conditions. Moreover, it has been suggested that this drug may modulate the pathological angiogenesis mediated by *Bartonella* spp [76].. Doxycycline should also be considered as a valid treatment option since there were no differences in relapses between the use of erythromycin and this drug [24]. However, this finding refers to PLWH and no data are available in patients without HIV infection or not suffering from the other major immunocompromising conditions listed above. By contrast, several failures and relapses were reported after treatment with aminoglycosides, trimethoprim/sulfamethoxazole and β-lactam antibiotics in angioproliferative lesions due to *Bartonella* spp [27].. As emerged from this analysis including patients not suffering from major immunocompromising conditions, a single-drug regimen administered for 42 (IQR: 14–47) days could be effective in treating a single cBA lesion, while multiple cBA lesions could require longer courses. This finding suggests that both host immune status and clinical features of cutaneous lesions (i.e., single or multiple) should be taken into consideration when prescribing treatment of cBA concerning type and duration. Indeed, longer antibiotic course and/or combined antimicrobial treatment is the standard of care in PLWH affected by cBA [24] as well as in other cutaneous infectious diseases [77]. However, this hint must be validated by further studies as for cBA concern since we were not able to conclude which is the best available treatment or whether the shorter therapy is more effective in these patients. Moreover, in cases with only a single lesion it could be interesting to know whether the treatment was motivated to prevent future dissemination of the disease, or by a persistent, remaining lesion. Unfortunately, we do not have enough data to address this intriguing question, as it was not answered by the literature that we have retrieved for the purpose of this review. However we suspect that the rationale for treatment may have been both to prevent future dissemination and treat persistent/remaining lesions.

Lastly, global warming is becoming a driver of major health problems in Europe, where the average air temperature has recently risen by one degree Celsius more than in the other continents [78]. In fact, several arthropod-borne infectious diseases such as Chikungunya, Dengue, West Nile and Zika are associated with global warming [78] and also *Bartonella* spp. incidence could be influenced by climate changes [79]. In particular, *Bartonella* spp. is susceptible to human intervention on environment and a selective pressure on vectors makes bartonellosis a possible re-emerging disease [2]. For instance, most studied vectors such as *Ctenocephalides felis* prefer warm climates (optimal temperature between 27 °C and 32 °C) [80]. Moreover, pets living in close contact with humans such as dogs and cats from warm countries have both a higher number of potential vectors and levels of bacteremia [46, 81, 82]. Therefore, the wide variety of *Bartonella* spp [75]., of pets as reservoirs [46, 81, 82] and of vectors [3, 38, 39] involved in human pathology as well as the recent climate changes make the prevalence of cBA highly dynamic and complex. Indeed, our review shows that cBA was mostly reported in warmer countries (India, Turkey, United States of America), while in Europe only three cases of cBA in patients not suffering from major immunocompromising conditions were reported [8, 21, 36]. In these countries, recent climate change could explain the emergence of cBA since it was not previously reported, although it cannot be excluded that the recent increase was due to more frequent diagnoses subsequent to the greater awareness of the problem and to a more widespread use of molecular biology techniques [12, 75]. To this regard, since PCR method allows to perform diagnosis using several samples, (such as frozen, paraffin embedded and lymph node tissues [83]), researchers should implement high-throughput sensitive techniques to identify *Bartonella* spp. DNA in line with what has been developed for other arthropod-born infections, by combining a broad-range PCR amplification of highly-conserved DNA regions (i.e., gene encoding the 16 S rRNA) with temporal temperature gradient gel electrophoresis [84]. However, despite preanalytical factors can hinder assay yield since formalin-fixed and paraffin-embedded tissues are less suitable than fresh-frozen ones for molecular diagnostic purpose [75], sequencing of specie-specific genes (i.e. 16–23 S rRNA internal transcribed spacer and citrate synthase regions) followed by molecular analysis can improve the routine data and identify serotype/genotype of *Bartonella* strains [85, 86]. This technique will likely be applied in high-income countries to help clinicians in diagnosis and follow-up of *Bartonella* spp. infections.

In summary, this review confirmes that cBA appears to be very rare in patients not suffering from major immunocompromising conditions but it also probably remains underdiagnosed due to the limited availability of molecular tests. However, in the era of next-generation sequencing, an active surveillance of re-emerging pathogens needs to be improved using molecular testing [87, 88]. Moreover, it is not possible to conclude which is the best available treatment or whether the shorter therapy is more effective in these patients. Indeed, the patients retrieved were very heterogeneous in terms of age and in several cases comorbidities were not reported, implying that further studies should be put in place to obtain more representative cohorts. Finally, in most of the cases reported the diagnosis of cBA was made by histological examination, while culture or PCR was performed in a minority of cases [8, 22, 27]. In this regard, despite cBA and verruga peruana are angioproliferative lesions that are not clinically or histopathologically distinguishable [23], only one case retrieved from the literature was reported in a Country where verruga peruviana is known to be endemic [34]. This observation further underscores the crucial role of species-specific PCR in facilitating species diagnosis. This method should be standardized and widely adopted for both diagnosis and treatment monitoring, potentially elevating the accuracy and efficiency of patient care across a variety of clinical settings [87, 88]..

## Conclusions

Since both *B. quintana* and *B. henselae* are responsible for a wide variety of cBA lesions which can be mistaken for similar ones due to others causes, *Bartonella* spp. are difficult to culture and PCR is not widely available in all settings, diagnosis is challenging and the burden of cBA in non severely immunocompromised patients could be overlooked. Therefore, the implementation of molecular testing is a necessary high-sensitivity test that could enable to treat and uncover the real burden in such cases. In conclusion, clinicians should consider cBA as a possible clinical manifestation of *B. quintana* or *B. henselae* infection even in patients not suffering from major immunocompromising conditions. Erythromycin should be considered the first choice while doxycycline and clarithromycin are valid alternatives. Rifampin may be useful in combination in some difficult to treat cases. A median antibiotic course of 42 and 90 days could be effective in single and multiple cBA lesions, respectively, but studies including more patients are needed to assess which is the most appropriate therapy for cBA. This is another piece of evidence that more attention should be given to a one health approach for prevention of infectious diseases in the current World.

## Data Availability

No datasets were generated or analysed during the current study.

## Abbreviations

cBA: cutaneous bacillary angiomatosis
HIV: human immunodeficiency virus
PLWH: people living with HIV
NIH: National Institute of Health
PCR: polymerase chain reaction
TLR: toll-like receptor
